# A new species of inseminating seasonal killifish of the *Cynopoecilus melanotaenia* complex from southern Brazil (Cyprinodontiformes: Rivulidae)

**DOI:** 10.3897/BDJ.4.e6888

**Published:** 2016-04-04

**Authors:** Wison JEM Costa, Pedro F Amorim, José Leonardo Oliveira Mattos

**Affiliations:** ‡ UFRJ - Universidade Federal do Rio de Janeiro, Rio de Janeiro, Brazil

**Keywords:** Aplocheiloid killifishes, Internal fertilization, Molecular phylogeny, Neotropical region, Systematics

## Abstract

**Background:**

The *Cynopoecilus melanotaenia* complex is a morphologically homogeneous killifish group, endemic from an area encompassing southern Brazil and northeastern Uruguay. It presently comprises four valid species: *C. melanotaenia*, the type species of the genus, and *C. fulgens*, *C. intimus*, and *C. nigrovittatus*.

**New information:**

*Cynopoecilus feltrini*, n. sp., from the lower Tubarão river basin, southern Brazil, is distinguished from all congeners of the *C. melanotaenia* complex by having frontal E-scales medially overlapped, branchiostegal region orangish red in males and dorsum with few dark brown spots above opercular region. A phylogenetic tree derived from the analysis of a fragment of the mitochondrial gene cytochrome c oxidase subunit I (681 bp) indicates that *C. feltrini* is a member of the clade that includes all species of the *C. melanotaenia* complex except *C. melanotaenia*, as well as that *C. feltrini* is the sister group of a clade comprising *C. fulgens* and *C. nigrovittatus*.

## Introduction

Killifishes of the tribe Cynopoecilini (Order Cyprinodontiformes, Family Rivulidae) comprise a well corroborated clade endemic of South America, between eastern Brazil and north-eastern Uruguay (Costa 2008, Costa 2014). They are members of the subfamily Cynolebiasinae, which uniquely includes seasonal species living in temporary pools (*e.g.*, Costa 2010). Two cynopoeciline genera, *Campellolebias* Vaz-Ferreira and Sierra 1974 and *Cynopoecilus* Regan 1912, are remarkable by containing the only internal fertilizing species of the suborder Aplocheiloidei (Vaz-Ferreira and Sierra 1974, Costa et al. 1989, Costa 1998).

*Cynopoecilus* is diagnosed, among other characters, by a morphological apparatus on the male anal fin related to insemination that is unique among killifishes (Costa 1995). It consists of a series of minute rays on the anterior part of the fin, forming an inseminating structure (Costa 1995, Costa 1998). In addition, *Cynopoecilus* may be easily diagnosed by the unique colour pattern occurring in all included species, consisting of a dark reddish brown to black stripe along the lateral midline of the body and another between the pectoral-fin base and the posterior end of the anal-fin base (Costa 1998, Costa 2002, Ferrer et al. 2014).

During the 90 years following its first description by Regan (1912), *Cynopoecilus* was considered as a monotypic subgenus of *Cynolebias* Steindachner 1876 (Myers 1952, Vaz-Ferreira and Sierra 1971, Parenti 1981), or as a monotypic genus (Costa 1990, Costa 1995), occurring in a vast area of southern Brazil and northeastern Uruguay (Vaz-Ferreira and Sierra 1971, Costa 1995). After larger collections made between 1997 and 1999, Costa (2002) provided the first taxonomical revision of *Cynopoecilus*, recognising five species: *C. melanotaenia* (Regan 1912), the type species of the genus, and *C. fulgens* Costa 2002, *C. intimus* Costa 2002, *C. multipapillatus* Costa 2002, and *C. nigrovittatus* Costa 2002; but a unpublished taxonomic revision of the genus by the first author indicates that *C. multipapillatus* is a synonym of *C. fulgens*. These species form a morphologically homogeneous group, herein called the *Cynopoecilus melanotaenia* complex, with all included species highly differing from another congener recently described by Ferrer et al. (2014), *C. notabilis* Ferrer et al. 2014. Among several diagnostic characters, species of that complex have the dorsal-fin origin in a vertical just anterior to anal-fin origin in males (*vs.* anterior to pelvic-fin insertion in *C. notabilis*), caudal fin rounded to sub-truncate in males (*vs.* lanceolate), unpaired fins hyaline in males, with pale spots or spots absent (*vs.* yellow with dark red spots), absence of a dark red stripe on the basal portion of the anal fin in males (*vs.* present), and flank in females without bars (*vs.* with bars).

More recently, collecting trips provided large new collections making possible a detailed revision of the *C. melanotaenia* complex, which is presently in progress. Among the new findings is a new species herein first described after evidence from morphology and mitochondrial DNA.

## Materials and methods

Material is deposited in the ichthyological collection of the Institute of Biology, Universidade Federal do Rio de Janeiro, Rio de Janeiro (UFRJ). Specimens were euthanized just after collection in a buffered solution of ethyl-3-amino-benzoat-methansulfonat (MS-222) at a concentration of 250 mg/l, for a period of 10 minutes or more, until completely ceasing opercular movements. Specimens fixed in formalin just after euthanasia for a period of 10 days, and then transferred to 70 % ethanol; specimens used in the molecular analysis were fixed in 98 % ethanol just after euthanasia and later preserved in the same fixative. List of specimens and respective GenBank accession numbers appear in Table 1.

Data on colour patterns were based numerous photographs of both sides of five live males and three live females, taken in aquaria between 1 and 78 hours after collection. Morphometric and meristic data were taken following Costa (1998); measurements are presented as percent of standard length (SL), except for those related to head morphology, which are expressed as percent of head length. Fin-ray counts include all elements. Number of vertebrae and gill-rakers were recorded from cleared and stained specimens; the compound caudal centrum was counted as a single element. Osteological preparations (c&s) were made according to Taylor and Dyke (1985). Terminology for frontal squamation follows Hoedeman (1958) and for cephalic neuromast series Costa (2001).

Total genomic DNA was extracted from muscle tissue of the right side of the caudal peduncle using the DNeasy Blood & Tissue Kit (Qiagen), according to the manufacturer instructions. To amplify the fragment of the DNA were used the primers L4299, H2198 (Folmer et al. 1994) and COX1F and COX1R (Costa and Amorim 2011), specific for the mitochondrial fragment of the cytochrome c oxidase subunit I (COX1). Polymerase chain reaction (PCR) was performed in 30μl reaction mixtures containing 5x Green GoTaq Reaction Buffer (Promega), 3.2 mM MgCl_2_, 1 μM of each primer, 75 ng of total genomic DNA, 0.2 mM of each dNTP and 1U of Taq polymerase. The thermocycling profile was: (1) 1 cycle of 4 minutes at 94°C; (2) 35 cycles of 1 minute at 92°C, 1 minute at 50°C and 1 minute and 30 seconds at 72°C; and (3) 1 cycle of 4 minutes at 72°C. In all PCR reactions, negative controls without DNA were used to check contaminations. Amplified PCR products were purified using the Wizard SV Gel and PCR Clean-Up System (Promega). Sequencing reactions were made using the BigDye Terminator Cycle Sequencing Mix (Applied Biosystems). Cycle sequencing reactions were performed in 10 μl reaction volumes containing 1 μl BigDye 2.5, 1.55 μl 5x sequencing buffer (Applied Biosystems), 2 μl of the amplified products (10–40ng), and 2 μl primer. The thermocycling profile was: (1) 35 cycles of 10 seconds at 96°C, 5 seconds at 54°C and 4 minutes at 60°C. The sequencing reactions were purified and denatured and the samples were run on an ABI 3130 Genetic Analyzer. Sequences were edited using MEGA 6 (Tamura et al. 2013) and aligned using ClustalW (Chenna et al. 2003).

Species were diagnosed using two criteria: unique combination of morphological character states (diagnosability criterion; *e.g.*, Davis and Nixon 1992) and DNA haplotype tree (*e.g.*, Wiens and Penkrot 2002). In the molecular phylogenetic analysis, terminal taxa included all species of the *C. melanotaenia* complex, except *C. intimus*, which was not found in recent collections due to deep habitat loss in the type locality area; outgroups were *C. notabilis*, the sister group of the *C. melanotaenia* complex; *Campellolebias brucei* Vaz-Ferreira and Sierra 1974, the type species of *Campellolebias*, the sister group of *Cynopoecilus*; and *Notholebias fractifasciatus* (Costa 1988), a member of *Notholebias*, a basal genus of the Cynopoecilini. Sequences were aligned using Clustal W (Chenna et al. 2003), after which the DNA sequences were translated into amino acids residues with MEGA 6.0 to test for the absence of premature stop codons or indels. The dataset was partitioned according to each codon position. The best fitting evolutionary model of each partition using Akaike information criteria (AIC) was determined with the software jModeltest version 2.1.7 (Darriba et al. 2012), respectively finding the models K80, F81 and GTR+I+G. The phylogenetic analyses were performed using Bayesian inference (BI) and maximum parsimony (MP) methods. BI analysis was conducted using MrBayes v3.2.5 (Ronquist et al. 2012) with the following settings: two Markov chain Monte Carlo (MCMC) runs of two chains each for 3 million generations, a sampling frequency of 100. All parameters between partitions except topology and branch lengths were unlinked. The appropriate burn-in fraction and convergence of the MCMC chains were graphically assessed by evaluating the stationary phase of the chains using Tracer v. 1.5 (Rambaut et al. 2013). The final consensus tree and Bayesian posterior probabilities (PP) were generated with the remaining tree samples after discarding the first 25% of samples as burn-in. The MP analysis was conducted with the software TNT 1.1 (Goloboff et al. 2008). To estimate the most parsimonious tree, traditional search was run with 100 trees saved per replication by tree bisection reconnection algorithm (TBR); 100 nonparametric bootstrap pseudo-replications were performed with the same software and a strict consensus tree was generated.

## Taxon treatments

### Cynopoecilus feltrini

Costa, Amorim & Mattos 2016 sp. n.

urn:lsid:zoobank.org:act:58847372-FF02-4483-8A4F-452F46920299

#### Materials

- **Type status:** Holotype. **Location:** country: Brazil; stateProvince: Santa Catarina; county: Laguna; locality: temporary pool near the confluence of Tubarão river and the Santo Antônio lagoon; verbatimElevation: 5 m; verbatimLatitude: 28°30'26"S; verbatimLongitude: 48°48'01"W; verbatimCoordinateSystem: degrees minutes seconds; verbatimSRS: Córrego Alegre; **Event:** year: 2015; month: 6; day: 10; habitat: Temporary pool; fieldNotes: collectors = C. Feltrin et al.; **Record Level:** institutionCode: UFRJ; collectionCode: 10662; basisOfRecord: PreservedSpecimen; dynamicProperties: sex=male, SL= 45.6 mm
- **Type status:** Paratype. **Record Level:** institutionID: UFRJ; collectionID: 10597; basisOfRecord: Preserved Specimen; dynamicProperties: 11 males, 18.2–48.0 mm SL, 11 females, 23.8–35.1 mm SL. Collected with holotype.
- **Type status:** Paratype. **Record Level:** institutionCode: UFRJ; collectionCode: 10598; basisOfRecord: Preserved Specimen; dynamicProperties: 3 males, 32.3–35.9 mm SL, 3 females, 22.5–34.1 mm SL. Collected with holotype.
- **Type status:** Paratype. **Record Level:** institutionCode: UFRJ; collectionCode: 10482; basisOfRecord: Preserved Specimen; dynamicProperties: 3 males, 20.3–32.2 mm SL, 2 females, 24.7–25.2 mm SL. Collected with holotype.
- **Type status:** Paratype. **Event:** year: 2015; month: 6; day: 4; **Record Level:** institutionCode: UFRJ; collectionCode: 10620; basisOfRecord: Cleared and Stained; dynamicProperties: 5 males, 27.9–46.9 mm SL, 2 females, 25.6–32.7 mm SL. Same locality of holotype.

#### Description

Morphometric data appear in Table 2. Largest male examined 48.0 mm SL; largest female examined 35.3 mm SL. Dorsal and ventral profiles slightly convex between snout and posterior end of dorsal and anal fins, nearly straight on caudal peduncle Fig. 1. Body slender, greatest body depth in vertical through pelvic-fin insertion. Urogenital papilla wide, with transverse opening projected over anal-fin origin. Longitudinal series of scales 27–28; transverse series of scales 9; scale rows around caudal peduncle 12. Contact organs on scales of caudal peduncle in males. Total vertebrae 29–30.

**Figure 2.**
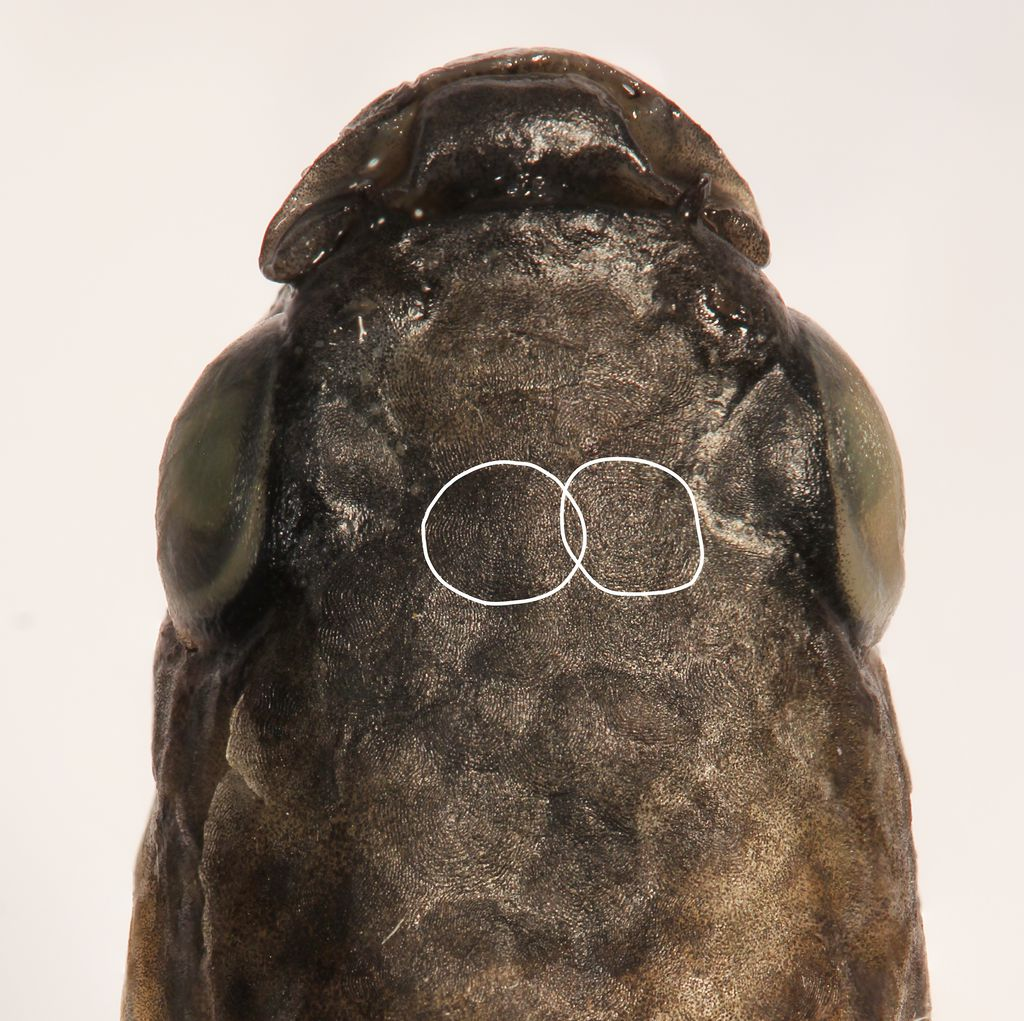
*Cynopoecilus feltrini* holotype dorsal view of the head, white circles highlight E-scales borders overlapped.

Eye positioned on dorsal portion of head side. Snout short, blunt. Premaxilla and dentary teeth conical, small, numerous, irregularly arranged, except for external series with longer fang-like teeth, slightly more robust in males. Vomerine teeth absent. Dermosphenotic absent. Frontal squamation usually E-patterned, sometimes D-patterned; E-scales often overlapping medially Fig. 2. Cephalic neuromasts: supraorbital 3 + 10 + 1, parietal 2, anterior rostral 1, posterior rostral 1, infraorbital 2–3 + 19–24, preorbital 4–6, otic 1, post-otic 2, supratemporal 1, pre-opercular 17–18, median opercular 1, ventral opercular 2–3, mandibular 8–10, lateral mandibular 7, paramandibular 1. Gill-rakers on first branchial arch 2 + 9. Six branchiostegal rays.

Dorsal and anal fins pointed in males, rounded in females; caudal fin rounded in both sexes; often short filamentous ray on tip of dorsal and anal fins, and minute posterior filamentous extension on middle of caudal fin in males. No scales on dorsal and anal fins, scales extending on about 30 % of caudal fin. Four to six neuromasts on caudal-fin base. Pectoral fin rounded, posterior margin reaching vertical between anus and urogenital opening in males, shorter, not reaching pelvic-fin base in females. No scales on pectoral-fin base. Pelvic-fin small, tip reaching between anus and urogenital opening in males, not reaching anus in females; pelvic-fin bases medially in close proximity. Dorsal-fin origin in vertical just anterior to anal-fin origin. Dorsal-fin origin between neural spines of vertebrae 12 and 13; anal-fin origin between pleural ribs of vertebrae 10 and 12 in males, between pleural ribs of vertebrae 12 and 13 in females. Hypurals forming single plate. Ventral process of posttemporal absent. Dorsal-fin rays 18–19; anal-fin rays 25–27; caudal-fin rays 29–32; pectoral-fin rays 13–15; pelvic-fin rays 5–7. No contact organ on fins.

Colouration. Males: Side of body pale brown to light yellowish brown, lighter on ventral portion; broad dark reddish brown to black stripe between posterior orbital margin and caudal-fin base, other similar narrower stripe between pectoral-fin base and posterior portion of anal-fin base; longitudinal rows of greenish blue to greenish golden spots, consisting of one spot per scale, on head side and flank, sometimes interrupted or rudimentary. Dorsum pale brown, with few dark brown spots above opercular region. Ventral portion of head and venter white. Lower jaw dark reddish brown. Few dark reddish brown on suborbital region. Branchiostegal membrane orangish red. Iris yellow, with dark reddish brown bar on middle, anterior and posterior portion with greenish golden iridescence. Unpaired fins pale grey; small dark grey spots on basal region of dorsal fin; light blue iridescence on margins of caudal and anal fins. Pectoral fin hyaline. Pelvic fin pale grey.

Females: Colour pattern similar to that described for males, but iridescent colour paler, median stripe often forming row of dark brown or black blotches, and faint grey dots present on basal portion of anal fin.

#### Diagnosis

Distinguished from all other species of the *Cynopoecilus melanotaenia* complex by having frontal E-scales medially overlapped (*vs.* separated by interspace), branchiostegal region orangish red in males (*vs.* hyaline to pinkish hyaline), dorsum with few dark brown spots above opercular region (*vs.* dark brown spots over most region between snout and dorsal-fin origin).

#### Etymology

Named after Caio Feltrin, in recognition of his dedication in inventorying the fish fauna of southern Brazil.

#### Distribution

**Figure 3.**
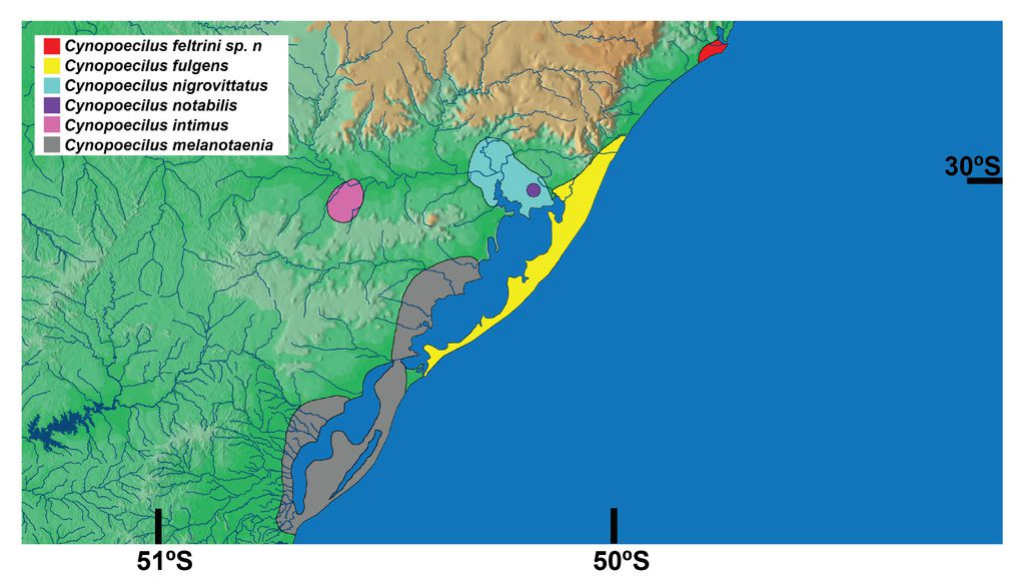
Distribution of *Cynopoecilus* species. Source: Costa (2002), Ferrer et al. (2014) and the current study for *C. feltrini*.

Known only from the type locality area, in temporary pools in the floodplains of the Tubarão river, Santa Catarina state, southern Brazil, corresponding to the northern-most record for the genus *Cynopoecilus* (Fig. 3).

#### Taxon discussion

*Cynopoecilus feltrini* is easily identifiable by some morphological characters. Among them, the frontal squamation pattern consisting of E-scales medially overlapped is unique among cynopoeciline killifishes, which have been diagnosed by the E-scales medially separated by an interspace (Costa 1998). Another striking feature of *C. feltrini* is the orangish red branchiostegal region in males, a condition not found in other congeners of the *C. melanotaenia* complex, but similar to the red branchiostegal region of *C. notabilis* (Ferrer et al. 2014). Finally, all species of the *C. melanotaenia* complex may be easily recognised among other cynopoecilines by the presence of numerous dark brown spots along the dorsum, but in *C. feltrini* these spots are restricted to region above the opercular region.

**Figure 4.**
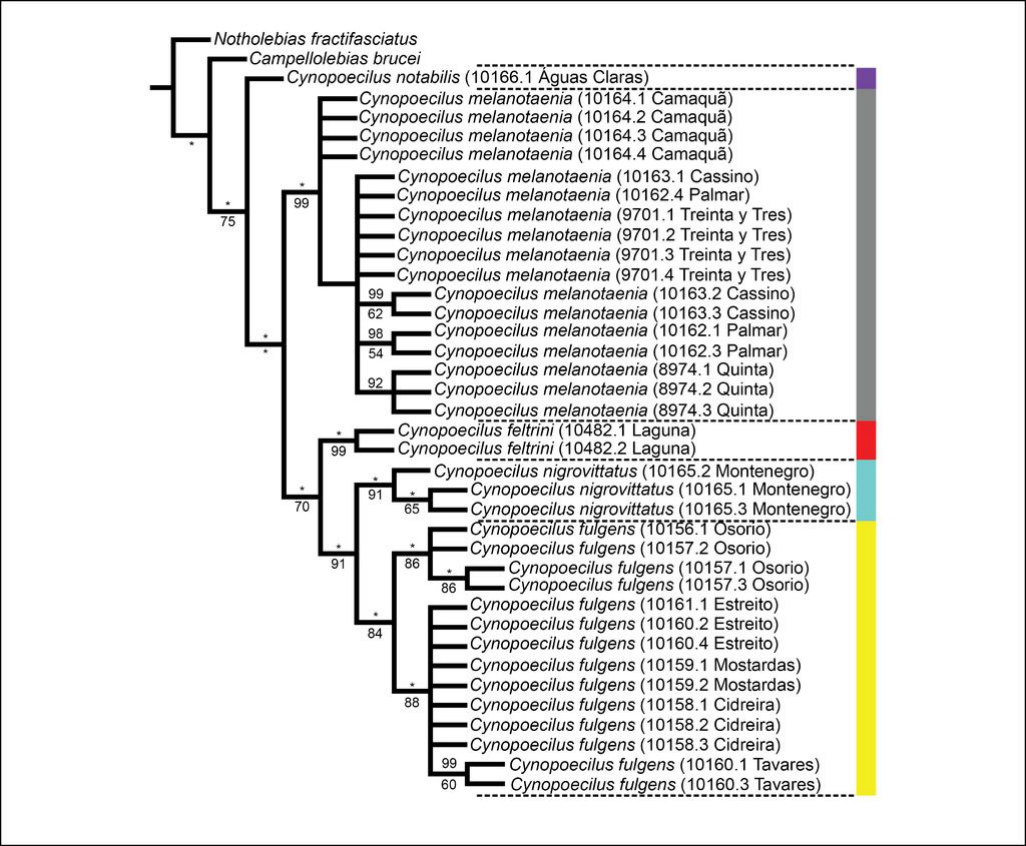
Maximum parsimony tree obtained from the COXI sequences. Numbers below the nodes are referring to the value of Bootstrap test and over the nodes are  the posterior probability of the Bayesian inference analysis, asterisks mean value of 100. Sequences used to generate the trees are listed in Table 1. Adjacent color bar indicates the species distribution area within shown in Fig. 3.

*Cynopoecilus feltrini* is presently known only from the lower Tubarão river basin, which is the northern-most record of the genus. The phylogenetic tree supports *C. feltrini* as a member of the clade that includes all species of the *C. melanotaenia* complex except *C. melanotaenia*, which is the taxon endemic of an area corresponding to the southern-most region of the genus distribution (Figs 3, 4). The analysis also supports sister-group relationships between *C. feltrini* and a clade comprising *C. fulgens*, from the coastal plains between the Patos lagoon and the sea, and *C. nigrovittatus*, from the lower Jacuí river drainage (Figs 3, 4). In addition, all the lineages corresponding to the four species of the *C. melanotaenia* complex sampled in this study had high bootstrap values in the analysis, this corroborating morphological data (Costa 2002, the present study). *Cynopoecilus intimus*, endemic of the upper Jacuí river basin, still has its phylogenetic position undetermined, since it was the only species of the *C. melanotaenia* complex not sampled for molecular data and morphological data alone do not provide unambiguous evidence about its position. In addition to diagnostic characters distinguishing *C. feltrini* from all other congeners discussed above, *C. feltrini* is easily distinguished from *C. intimus* by the latter having large dark brown blotches on the flank immediately below the dorsal-fin base (*vs.* blotches absent in *C. feltrini*) and having minute pelvic fin in males, pelvic-fin length 4.7–5.6 % SL, its tip reaching anus or shorter (*vs.* 5.9–7.3 % SL in *C. feltrini*, pelvic-fin tip reaching space between anus and urogenital opening).

### Notholebias fractifasciatus

Costa, 1998

#### Materials

- **Type status:** Other material. **Location:** country: Brazil; municipality: Inoã; verbatimLatitude: 22°55'21"S; verbatimLongitude: 42°55'42"W; **Record Level:** datasetID: 8802; institutionCode: UFRJ

#### Notes

This taxon was included as terminal in phylogenetic analysis of this study.  Genbank access code to the sequences in Table 1

### Campellolebias brucei

Vaz-Ferreira & Sierra de Soriano, 1974

#### Materials

- **Type status:** Other material. **Location:** country: Brazil; stateProvince: Santa Catarina; county: Florianópolis; verbatimLatitude: 27°40'59"S; verbatimLongitude: 48°33'38"W; verbatimSRS: Córrego Alegre; **Record Level:** datasetID: 8383; institutionCode: UFRJ; basisOfRecord: PreservedSpecimen

#### Notes

This taxon was included as terminal in phylogenetic analysis of this study.  Genbank access code to the sequences in Table 1​

### Cynopoecilus melanotaenia

(Regan, 1912)

#### Materials

- **Type status:** Other material. **Location:** country: Brazil; stateProvince: Rio Grande do Sul; county: Quinta; verbatimLatitude: 32°04'13"S; verbatimLongitude: 52°15'49"W; **Record Level:** datasetID: 8974; institutionCode: UFRJ
- **Type status:** Other material. **Location:** country: Uruguay; stateProvince: Treinta y Tres; verbatimLatitude: 32°45'57"S; verbatimLongitude: 53°44'09"W; **Record Level:** datasetID: 9701; institutionCode: UFRJ
- **Type status:** Other material. **Location:** country: Brazil; stateProvince: Rio Grande do Sul; municipality: Palmar; verbatimLatitude: 32°44'40"S; verbatimLongitude: 52°38'41"W; **Record Level:** datasetID: 10162; institutionCode: UFRJ
- **Type status:** Other material. **Location:** country: Brazil; stateProvince: Rio Grande do Sul; county: Cassino; verbatimLatitude: 32°06'00"S; verbatimLongitude: 52°09'55"W; **Record Level:** datasetID: 10163; institutionCode: UFRJ
- **Type status:** Other material. **Location:** country: Brazil; stateProvince: Rio Grande do Sul; county: Camaquã; verbatimLatitude: 31°04'41"S; verbatimLongitude: 52°02'18"W; **Record Level:** datasetID: 10164; institutionCode: UFRJ

#### Notes

This taxon was included as terminal in phylogenetic analysis of this study.  Genbank access code to the sequences in Table 1​

### Cynopoecilus fulgens

Costa, 2002

#### Materials

- **Type status:** Other material. **Location:** country: Brazil; stateProvince: Rio Grande do Sul; county: Osório; verbatimLatitude: 29°57'34"S; verbatimLongitude: 50°13'53"W; **Record Level:** datasetID: 10156; institutionCode: UFRJ
- **Type status:** Other material. **Location:** country: Brazil; stateProvince: Rio Grande do Sul; county: Cidreira; verbatimCoordinates: 30°09'09"S, 50°14'25"W; **Record Level:** datasetID: 10158; institutionCode: UFRJ
- **Type status:** Other material. **Location:** country: Brazil; stateProvince: Rio Grande do Sul; county: Mostardas; verbatimCoordinates: 30°50'59"S, 50°41'21"W; **Record Level:** datasetID: 10159; institutionCode: UFRJ
- **Type status:** Other material. **Location:** country: Estreito; stateProvince: Rio Grande do Sul; county: Estreito; verbatimCoordinates: 31°15'52"S, 51°43'31"W; **Record Level:** datasetID: 10160; institutionCode: UFRJ

#### Notes

This taxon was included as terminal in phylogenetic analysis of this study.  Genbank access code to the sequences in Table 1​

### Cynopoecilus nigrovittatus

Costa, 2002

#### Materials

- **Type status:** Other material. **Location:** country: Brazil; stateProvince: Rio Grande do Sul; county: Montenegro; verbatimCoordinates: 29°40'12"S, 51°25'32"W; **Record Level:** datasetID: 10165; institutionCode: UFRJ

#### Notes

This taxon was included as terminal in phylogenetic analysis of this study.  Genbank access code to the sequences in Table 1​

### Cynopoecilus notabilis

Ferrer, Wingert & Malabarba, 2014

#### Materials

- **Type status:** Other material. **Location:** country: Brazil; stateProvince: Rio Grande do Sul; county: Águas Claras; verbatimCoordinates: 30°05'48"S, 50°51'06"W; **Record Level:** datasetID: 10166; institutionCode: UFRJ

#### Notes

This taxon was included as terminal in phylogenetic analysis of this study.  Genbank access code to the sequences in Table 1​

## Supplementary Material

XML Treatment for Cynopoecilus feltrini

XML Treatment for Notholebias fractifasciatus

XML Treatment for Campellolebias brucei

XML Treatment for Cynopoecilus melanotaenia

XML Treatment for Cynopoecilus fulgens

XML Treatment for Cynopoecilus nigrovittatus

XML Treatment for Cynopoecilus notabilis

## Figures and Tables

**Figure 1a.**
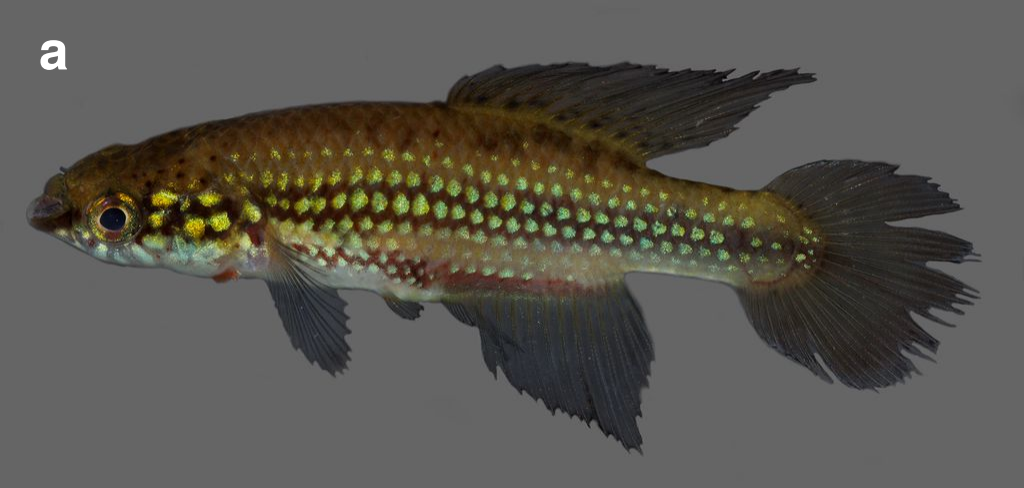
UFRJ 10662, holotype, male, 45.6 mm SL.

**Figure 1b.**
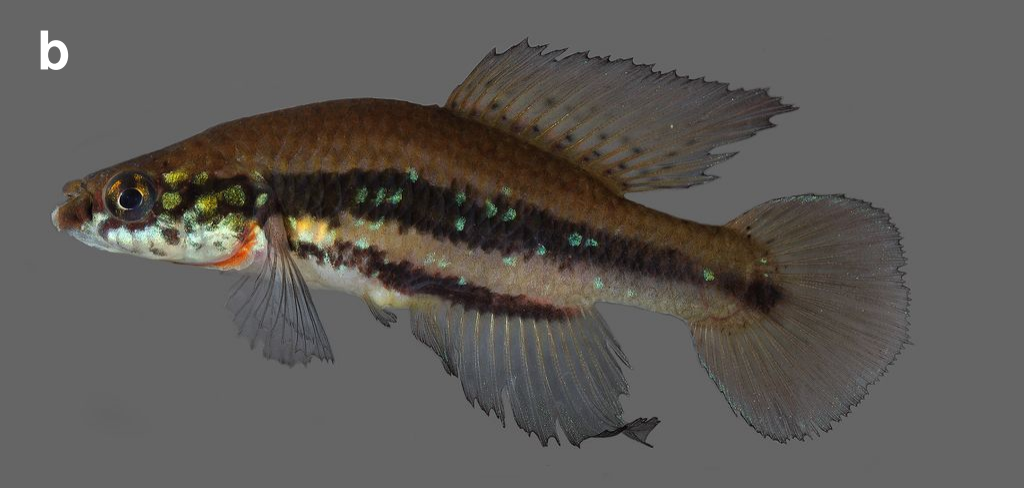
UFRJ 10597, paratype, male, 43.5 mm SL.

**Figure 1c.**
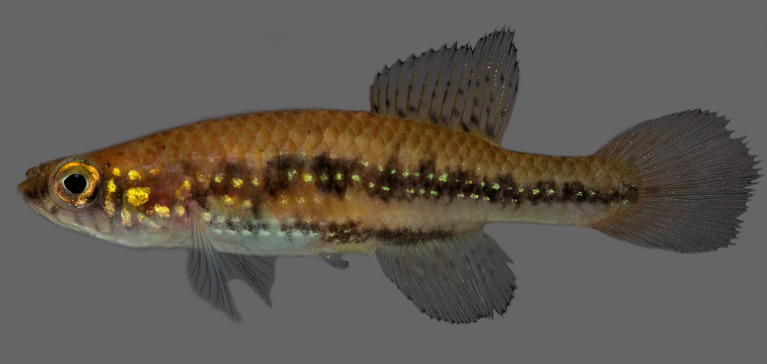
UFRJ 10597, paratype, female, 35.3 mm SL.

**Table 1.** List of species, localities and respective catalogue numbers and GenBank accession numbers.

**Species**	**Catalog number**	**Locality**	**GenBank (COX1)**
*Notholebias fractifasciatus*			
	UFRJ 8802.1	Inoã	KT590062
		(22°55'21"S, 42°55'42"W)	
*Campellolebias brucei*			
	UFRJ 8383	Florianópolis	KT590065
		(27°40'59"S, 48°33'38"W)	
*Cynopoecilus melanotaenia*			
	UFRJ 8974.1	Quinta	KT590066
		(32°04'13"S, 52°15'49"W)	
	UFRJ 8974.2	Quinta	KT823646
		(32°04'13"S, 52°15'49"W)	
	UFRJ 8974.3	Quinta	KT823647
		(32°04'13"S, 52°15'49"W)	
	UFRJ 9701.1	Treinta y Tres	KT823648
		(32°45'57"S, 53°44'09"W)	
	UFRJ 9701.2	Treinta y Tres	KT823649
		(32°45'57"S, 53°44'09"W)	
	UFRJ 9701.3	Treinta y Tres	KT823650
		(32°45'57"S, 53°44'09"W)	
	UFRJ 9701.4	Treinta y Tres	KT823651
		(32°45'57"S, 53°44'09"W)	
	UFRJ 10162.1	Palmar	KT823665
		(32°44'40"S, 52°38'41"W)	
	UFRJ 10162.3	Palmar	KT823666
		(32°44'40"S, 52°38'41"W)	
	UFRJ 10162.4	Palmar	KT823667
		(32°44'40"S, 52°38'41"W)	
	UFRJ 10163.1	Cassino	KT823668
		(32°06'00"S, 52°09'55"W)	
	UFRJ 10163.2	Cassino	KT823669
		(32°06'00"S, 52°09'55"W)	
	UFRJ 10163.3	Cassino	KT823670
		(32°06'00"S, 52°09'55"W)	
	UFRJ 10164.1	Camaquã	KT823671
		(31°04'41"S, 52°02'18"W)	
	UFRJ 10164.2	Camaquã	KT823672
		(31°04'41"S, 52°02'18"W)	
	UFRJ 10164.3	Camaquã	KT823673
		(31°04'41"S, 52°02'18"W)	
	UFRJ 10164.4	Camaquã	KT823674
		(31°04'41"S, 52°02'18"W)	
*Cynopoecilus fulgens*			
	UFRJ 10156.1	Osório	KT823652
		(29°57'34"S, 50°13'53"W)	
	UFRJ 10157.1	Osório	KT823653
		(29°59'20"S, 50°11'32"W)	
	UFRJ 10157.2	Osório	KT823654
		(29°59'20"S, 50°11'32"W)	
	UFRJ 10157.3	Osório	KT823655
		(29°59'20"S, 50°11'32"W)	
	UFRJ 10158.1	Cidreira	KT823656
		(30°09'09"S, 50°14'25"W)	
	UFRJ 10158 2	Cidreira	KT823657
		(30°09'09"S, 50°14'25"W)	
	UFRJ 10158.3	Cidreira	KT823658
		(30°09'09"S, 50°14'25"W)	
	UFRJ 10159.1	Mostardas	KT823659
		(30°50'59"S, 50°41'21"W)	
	UFRJ 10159.2	Mostardas	KT823660
		(30°50'59"S, 50°41'21"W)	
	UFRJ 10160.1	Estreito	KT590069
		(31°15'52"S, 51°43'31"W)	
	UFRJ 10160.2	Estreito	KT823661
		(31°15'52"S, 51°43'31"W)	
	UFRJ 10160.3	Estreito	KT823662
		(31°15'52"S, 51°43'31"W)	
	UFRJ 10160.4	Estreito	KT823663
		(31°15'52"S, 51°43'31"W)	
	UFRJ 10161.1	Estreito	KT823664
		(31°49'18"S, 51°43'31"W)	
*Cynopoecilus nigrovittatus*			
	UFRJ 10165.1	Montenegro	KT590067
		(29°40'12"S, 51°25'32"W)	
	UFRJ 10165.2	Montenegro	KT823675
		(29°40'12"S, 51°25'32"W)	
	UFRJ 10165.3	Montenegro	KT823676
		(29°40'12"S, 51°25'32"W)	
*Cynopoecilus notabilis*			
	UFRJ 10166	Aguas Claras	KT590068
		(30°05'48"S, 50°51'06"W)	
*Cynopoecilus feltrini*			
	UFRJ 10482.1	Laguna	KT823677
		(28°30'42"S, 48°47'59"W)	
	UFRJ 10482.2	Laguna	KT823678
		(28°30'42"S, 48°47'59"W)	

**Table 2.** Morphometric data of *Cynopoecilus feltrini*.

	**holotype**	**paratypes**	
	**male**	**males (9)**	**females (5)**
Standard length (mm)	45.6	32.3–48.0	26.5–35.3
**Percent of standard length**			
Body depth	28.2	27.4–32.0	27.2–32.3
Caudal peduncle depth	13.8	12.8–15.0	12.1–14.1
Pre-dorsal length	54.3	53.4–57.4	59.6–62.8
Pre-pelvic length	48.6	45.4–49.6	51.6–53.9
Length of dorsal-fin base	28.8	26.0–30.4	24.5–26.4
Length of anal-fin base	27.1	25.4–27.9	20.0–22.4
Caudal-fin length	34.1	30.9–35.2	30.8–33.1
Pectoral-fin length	19.1	20.0–22.5	19.4–21.5
Pelvic-fin length	6.5	5.9–7.3	5.5–7.0
Head length	27.9	27.6–30.2	27.2–29.8
**Percent of head length**			
Head depth	71.1	71.2–76.2	76.1–79.6
Head width	68.8	65.1–72.9	71.0–79.1
Snout length	14.3	12.7–14.7	12.7–14.1
Lower jaw length	18.7	16.9–19.4	16.5–18.5
Eye diameter	31.4	31.5–37.3	33.4–39.1
